# Ovarian pregnancy following fresh embryo transfer: A case report and literature review

**DOI:** 10.1016/j.crwh.2025.e00744

**Published:** 2025-08-19

**Authors:** Fombeno Kueta Pascal, Johnny El Hanna, Abdelilah Arsalane, Justin Lewis Denakpo

**Affiliations:** a Grand Hopital de l'Est Francilien, Meaux, Seine et Marne, France; b Université d'Abomey-Calavi Faculté des Sciences de la Santé, Republic of Benin

**Keywords:** Ovarian pregnancy, Ectopic pregnancy, Embryo transfer, In vitro fertilization, Assisted reproductive technology

## Abstract

Ovarian pregnancy is a rare and potentially life-threatening form of ectopic pregnancy, accounting for only 1.6 % to 2 % of all ectopic gestations. While its association with assisted reproductive technology is increasingly recognized, diagnosis remains challenging due to its nonspecific clinical and imaging features.

This report concerns the case of a 29-year-old primigravida who presented with mild pelvic pain and vaginal spotting following a fresh embryo transfer. Transvaginal ultrasound revealed an empty uterus and a right ovarian mass. The patient had a serum β-hCG level of 3952 IU/L. Diagnostic laparoscopy indicated the presence of an ectopic pregnancy within the right ovary. Histopathological analysis of the excised mass confirmed the diagnosis. The patient recovered uneventfully following surgical management.

Ovarian pregnancy following embryo transfer is an uncommon but serious complication of the use of assisted reproductive technology. Early diagnosis is crucial to prevent complications and preserve fertility.

## Highlights

• Ovarian pregnancy is a rare form of ectopic pregnancy, sometimes occurring after the use of assisted reproduction techniques.
• Early ultrasound evaluation is crucial for timely diagnosis.
• Surgical management remains the mainstay of treatment.

## 1 Introduction

Ovarian pregnancy is a rare form of ectopic pregnancy. It accounts for 1.6 % to 2 % of all ectopic pregnancies [1,2]. The main risk factors are a history of pelvic surgery and the use of intrauterine devices, present in 39.29 % and 18.75 % of cases respectively [3]. A growing and significant risk factor for ectopic pregnancies is the use of assisted reproductive technology, particularly embryo transfer techniques, which are complicated by ectopic pregnancies in 4.5 % of cases—of which 6 % are ovarian in location [4]. The occurrence of ovarian pregnancy after embryo transfer is explained by two etiopathogenic theories. The first is the theory of uterotubal reflux described by L. Iffy in 1963 [5]. The second relates to embryo transfer, whereby one or more embryos pass directly through the fallopian tubes via the intra-cervical catheter under transfer pressure.

Early diagnosis of ovarian pregnancy remains a challenge for medical teams, as it is most often made during a diagnostic laparoscopy, which is frequently combined with surgical management. A definitive diagnosis is established either through histological examination of the surgical specimen or during laparoscopy when a gestational sac with an embryo is clearly identified.

This report presents a case of an ovarian pregnancy that developed after fresh embryo transfer in a 29-year-old primigravida with no identifiable risk factors; additionally, the literature is reviewed.

## 2 Case Presentation

A 29-year-old woman with primary infertility due to anovulatory dysfunction presented to the gynecological emergency ward with pelvic pain and light vaginal bleeding at 5 weeks and 4 days of amenorrhea. She had undergone a fresh embryo transfer as part of in vitro fertilization (IVF) following a history of three failed ovulation induction cycles and two failed intrauterine insemination (IUI) cycles. Ovulation induction was performed using rhFSH 125 IU, with trigger administration using r-hCG, followed by a day-5 fresh embryo transfer. The patient reported no intercourse at the time of ovulation.

**Fig. 1:**
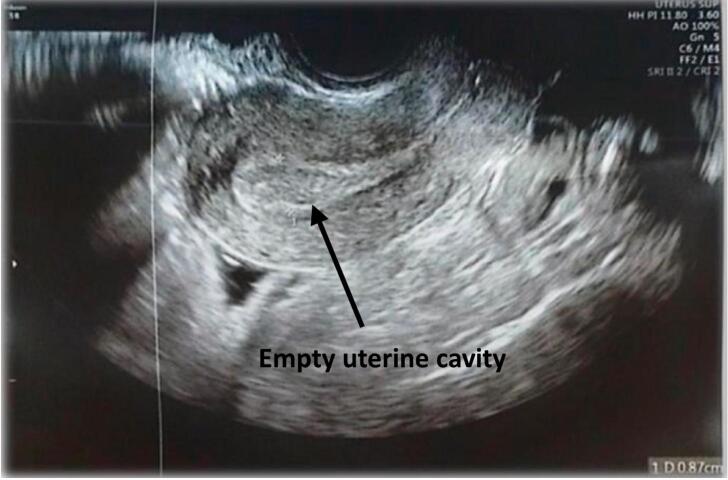
Transvaginal ultrasound showing an empty uterine cavity.

**Fig. 2:**
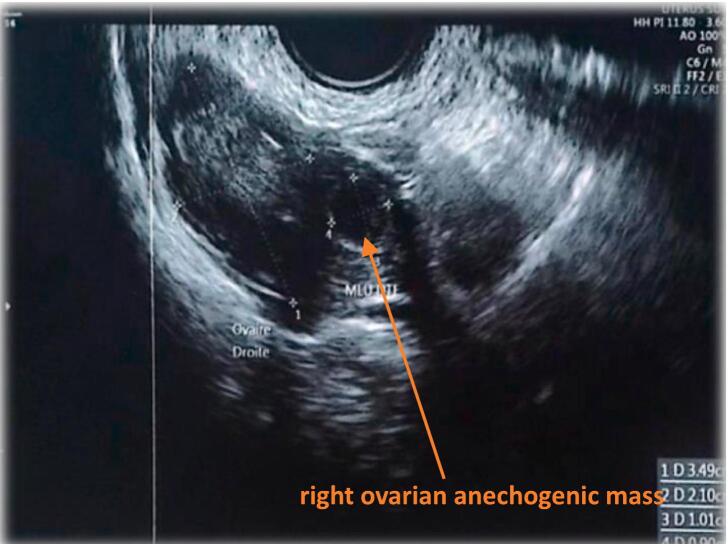
Transvaginal ultrasound showing the right ovarian mass.

The patient had no significant medical history and did not smoke. Her hysterosalpingography (HSG) was normal, ruling out any structural issues with the uterus or fallopian tubes. On examination, she exhibited mild suprapubic tenderness, cervical motion tenderness, and minimal vaginal bleeding. Ultrasound showed an empty uterus *(*Fig. 1*)*, 8 mm endometrial thickness, a right ovarian anechogenic mass measuring 12.5 mm × 11.3 mm *(*Fig. 2*)*, and minimal free fluid in the cul-de-sac. The β-HCG level was 3952 IU/L, indicating pregnancy. These clinical, biochemical, and ultrasonographic findings strongly suggest an ectopic pregnancy at the outset. However, the possibility of a hemorrhagic ovarian cyst or a corpus luteum cyst cannot be definitively excluded. Despite analgesics, her pain persisted, prompting the decision to proceed with diagnostic laparoscopy.

**Fig. 3:**
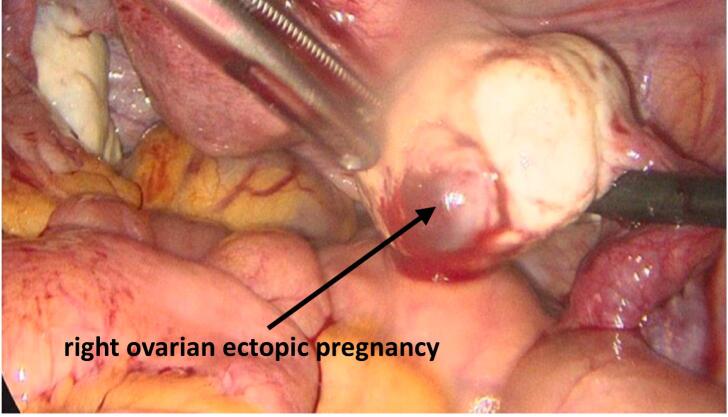
Intraoperative laparoscopic image showing the right ovarian ectopic pregnancy.

**Fig. 4:**
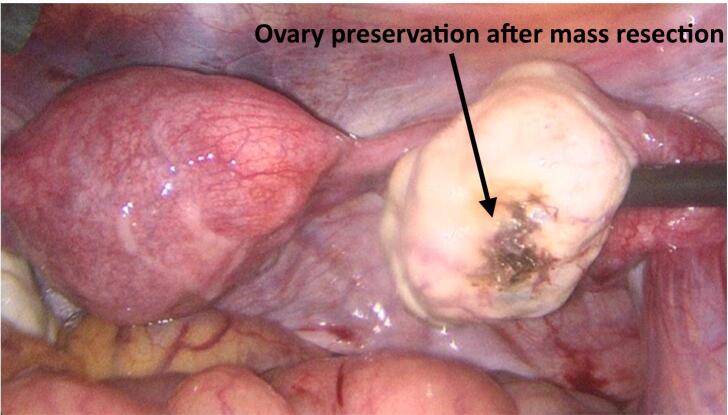
Intraoperative laparoscopic image showing the right ovary after resection.

During laparoscopy, minimal hemoperitoneum was noted, and a right ovarian mass was identified *(*Fig. 3*)*, which appeared to be on the verge of expulsion from the ovary. The fallopian tubes and uterus appeared normal. The mass was removed and placed in an endoscopic retrieval bag for histopathological examination, with preservation of ovarian tissue *(*Fig. 4*)*. The intraoperative blood loss was 150 mL.

**Fig. 5:**
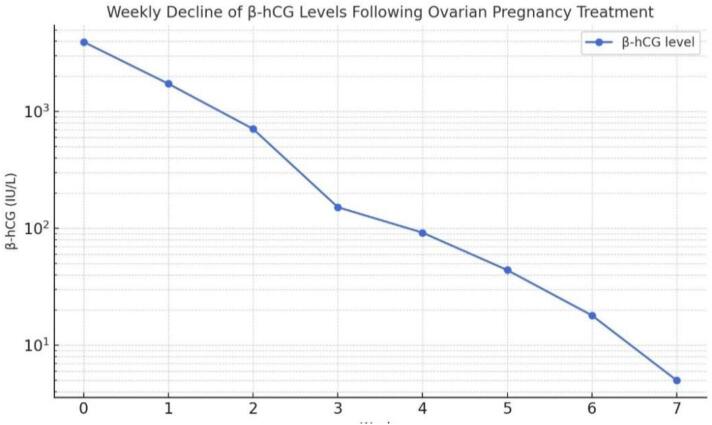
Weekly decline of β-hCG levels following Ovarian Pregnancy Treatment.

The patient tolerated the procedure well and remained stable postoperatively. The pathology report confirmed the presence of trophoblastic material, thereby confirming the diagnosis of an ovarian pregnancy. Postoperatively, β-HCG levels were monitored, showing a decline from 3952 IU/L to 1733 IU/L, 711 IU/L, 152 IU/L, 92 IU/L, 44 IU/L and finally <5 IU/L (Fig. 5), confirming the resolution of the pregnancy. The patient was advised to use a progestin-only contraceptive for the subsequent three cycles, with an ultrasound examination scheduled after three months to assess ovarian healing. Resumption of the IVF program might be considered thereafter, contingent upon the ovarian status.

## 3 Discussion

In France today, assisted reproductive technologies are largely dominated by in vitro fertilization and embryo transfer. The European Society of Human Reproduction and Embryology (ESHRE) recommends several treatment protocols that must be tailored to each patient's profile [6].

The present patient was young and had no significant medical history. She underwent an antagonist protocol with ovulation induction using 125 IU of recombinant FSH and ovulation triggered through HCG administration. She reported no sexual intercourse since the beginning of the protocol, effectively ruling out the possibility of a spontaneous pregnancy. The pregnancy was therefore clearly the result of embryo transfer.

The literature does not establish a causal link between the stimulation protocol used and the occurrence of ovarian pregnancy. However, the origin of the oocyte, whether it was obtained through donation or collected via follicular retrieval, appears to be an important factor. A 2016 Canadian study reported ovarian implantation at the needle puncture site used for oocyte aspiration [7].

Generally, ectopic pregnancy is suspected on the basis of a combination of serum β-hCG levels and pelvic ultrasound [8]. Ovarian pregnancy follows the same diagnostic algorithm. Clinical signs are similar to those of other ectopic pregnancies [9], most commonly pelvic pain (in 89.29 % of cases) and vaginal bleeding (in 41.07 % of cases) [3].

Pain in ovarian pregnancy is mainly due to rupture of the ovarian capsule and subsequent peritoneal effusion of varying severity [10]. In this case, the patient presented with mild pelvic pain and light vaginal bleeding, which were linked to a light tear rather than a full rupture of the ovarian capsule. A small amount of free fluid in the pouch of Douglas was observed, first via ultrasound and later confirmed during laparoscopy.

In the literature, the diagnostic value of transvaginal ultrasound for ovarian pregnancy is well established. Modern diagnostic approaches require criteria including: serum β-hCG ≥ 1000 IU/L, an empty uterus on transvaginal ultrasound, involvement of the affected ovary during surgical exploration, the presence of an atypical ovarian cyst, and a decline in β-hCG following treatment [12].

In a 2008 case, Plotti et al. [13] diagnosed bilateral ovarian pregnancy directly via ultrasound in a 34-year-old patient, based on the presence of a gestational sac in each ovary—each containing an embryo, with the left showing cardiac activity. A similar case was described by Han et al. [14], involving a 38-year-old woman whose ultrasound showed bilateral ovarian cysts without any adnexal masses, and an empty uterus with β-hCG at 62,520 IU/L. An endometrial biopsy ruled out intrauterine pregnancy, and exploratory laparotomy revealed that the cysts were in fact bilateral ovarian pregnancies, later confirmed by histology. This last case closely resembles the one in this report. The present patient's transvaginal ultrasound revealed an anechoic formation in the right ovary, no adnexal mass, and an empty uterus; her β-hCG level was 3952 IU/L. Laparoscopic exploration showed normal fallopian tubes and an ovarian mass that suggested a fissured right ovarian pregnancy, though a ruptured hemorrhagic cyst could not be completely excluded. It was ultimately the histological exam that confirmed the diagnosis of ovarian pregnancy.

In the literature, a similar case was reported in 2024 by Chen et al. [18], following a frozen embryo transfer in a 29-year-old patient with a history of a left ovarian ectopic cyst and bilateral tubal obstruction who had engaged in sexual intercourse during the transfer. That case involved a heterotopic pregnancy with a ruptured right tubal ectopic pregnancy accompanied by pelvic effusion. In the present case, the patient was of the same age, without relevant medical history, and reported no sexual activity during the transfer. Although her ectopic pregnancy was located differently (ovary versus tube), it occurred on the same side (right).

A reported in August 2025 by Ban et al. [11] involved a 38-year-old patient who developed a right tubal pregnancy following embryo transfer, treated by laparoscopic salpingectomy. During the procedure, the left tube appeared normal. Postoperative β-hCG monitoring revealed a contralateral (left) tubal pregnancy, which was also managed laparoscopically. In the present case, postoperative β-hCG levels progressively decreased, confirming the unilateral nature of the ectopic pregnancy.

Treatment of ectopic pregnancy includes several therapeutic options, each with specific indications. These typically include surgical treatment via laparoscopy, medical management with methotrexate, or in cases where β-hCG levels are below 1000 IU/L (with or without decreasing kinetics), an expectant approach is preferred [15]. Ovarian pregnancy, however, does not fully conform to this range of options. While Shamma and Schwartz [16] reported a successful methotrexate-treated case in 1992, a 2023 study by Mengyuan et al. [3] analyzed 112 cases of ovarian pregnancy, all of which ultimately required surgical treatment. Among them, 76 % underwent immediate surgery, 3 % after failure of methotrexate therapy, and 21 % after failed expectant management. In a study spanning 12 years, Arieh et al. [17] concluded that laparoscopic wedge resection remains the best treatment for ovarian pregnancy. Accordingly, the present patient underwent laparoscopic surgery from the outset, allowing both diagnosis and treatment in the same procedure.

Based on this case report, we recommend that an early targeted ultrasound after embryo transfer should be considered in patients with pelvic pain, even in the absence of high-risk factors.

## 4 Conclusion

Ovarian pregnancy remains a rare but serious form of ectopic pregnancy, with increasing relevance in the context of assisted reproductive technologies. Despite the absence of traditional risk factors, ovarian implantation can still occur, as demonstrated in this case. Early diagnosis remains difficult due to non-specific clinical signs and frequent confusion with hemorrhagic ovarian cysts. Surgical intervention, particularly via laparoscopy, continues to be the cornerstone of both diagnosis and management, especially when conservative or medical options are limited or contraindicated. We recommend that an early targeted ultrasound after embryo transfer should be considered in patients with pelvic pain, even in the absence of high-risk factors.

## Contributors

Fombeno Kueta Pascal contributed to patient care, conception of the case report, drafting the manuscript and undertaking the literature review.

Johnny El Hanna contributed to patient care, conception of the case report and drafting the manuscript.

Abdelilah Arsalane contributed to revising the article critically for important intellectual content.

Justin Lewis Denakpo contributed to revising the article critically for important intellectual content.

All authors approved the final submitted manuscript.

## Patient consent

Written informed consent was obtained from the patient for publication of the case report and accompanying images.

## Provenance and peer review

This article was not commissioned and was peer reviewed.

## Funding

The authors received no external funding for this work.

## Declaration of competing interest

The authors declare that they have no competing interest regarding the publication of this case report.
